# Rac1 Dynamics in the Human Opportunistic Fungal Pathogen *Candida albicans*

**DOI:** 10.1371/journal.pone.0015400

**Published:** 2010-10-28

**Authors:** Romain Vauchelles, Danièle Stalder, Thomas Botton, Robert A. Arkowitz, Martine Bassilana

**Affiliations:** Institute of Developmental Biology and Cancer, Centre National de la Recherche Scientifique UMR 6543, Université de Nice, Faculté des Sciences-Parc Valrose, Nice, France; Dartmouth College, United States of America

## Abstract

The small Rho G-protein Rac1 is highly conserved from fungi to humans, with approximately 65% overall sequence identity in *Candida albicans*. As observed with human Rac1, we show that *C. albicans* Rac1 can accumulate in the nucleus, and fluorescence recovery after photobleaching (FRAP) together with fluorescence loss in photobleaching (FLIP) studies indicate that this Rho G-protein undergoes nucleo-cytoplasmic shuttling. Analyses of different chimeras revealed that nuclear accumulation of *C. albicans* Rac1 requires the NLS-motifs at its carboxyl-terminus, which are blocked by prenylation of the adjacent cysteine residue. Furthermore, we show that *C. albicans* Rac1 dynamics, both at the plasma membrane and in the nucleus, are dependent on its activation state and in particular that the inactive form accumulates faster in the nucleus. Heterologous expression of human Rac1 in *C. albicans* also results in nuclear accumulation, yet accumulation is more rapid than that of *C. albicans* Rac1. Taken together our results indicate that Rac1 nuclear accumulation is an inherent property of this G-protein and suggest that the requirements for its nucleo-cytoplasmic shuttling are conserved from fungi to humans.

## Introduction

Proteins of the Rho GTPases family, which include Rac and Cdc42, function as molecular switches. They cycle between an inactive GDP-bound form and an active GTP-bound form, which interacts with downstream effectors to transduce signals. They are activated by guanine nucleotide exchange factors (GEFs) and inactivated by GTPase activating proteins (GAPs) and regulated by Rho GDP-dissociation inhibitors (GDIs). In humans, a total of 20 Rho GTPases are activated by more than 80 GEFs, which belong to two distinct families [1], [2], [3], and are inactivated by approximately 70 GAPs and regulated by 3 GDIs [4]. The yeast *Saccharomyces cerevisiae* has 6 Rho GTPases, yet homologs of Rac, which has been proposed to be the founder of the Rho GTPase family [5], are not present. Rac1 is, however, ubiquitously present in virtually all other eukaryotes from human to fungi, including in the human opportunistic pathogen *Candida albicans* [6].

In mammals, Rac1 regulates multiple signaling pathways that control a number of cellular functions, such as cell polarity or gene transcription [7]. The cellular localization of Rac1 is critical for specifying such diverse functions, *via* site-specific activation/inactivation and a range of protein interactions. Rac1 cycles between the plasma membrane, where it associates *via* geranylgeranylation of its carboxy-terminal cysteine residue [8] and the cytosol, where it is bound to RhoGDI [9]. Rac1 has also been shown to accumulate in the nucleus, where it was implicated in different functions, such as cell division [10], nuclear import of the transcription factor STAT5 [11], accumulation of the armadillo repeat protein smgGDS [12] and for its own proteasome-mediated degradation [13]. One essential feature for Rac1 localization is the presence of a carboxyl-terminal polybasic region (PBR), which contains a nuclear localization sequence (NLS) [14], preceded by three prolines [10]. Furthermore, both the Rac1 GEF, Dock180, together with the regulatory protein ELMO [15] and the Rac1 GAP, MgcRacGAP [16] have also been observed in the nucleus. Whether the active GTP-bound form or the inactive GDP-bound form of Rac1 accumulates differentially in the nucleus is however controversial [10], [12], [17], [18].

In fungi, Rac1 is also required for different functions such as hyphal differentiation, invasive growth and virulence [19], [20], [21], [22]. In *C. albicans*, Rac1 and its specific activator Dck1 are dispensable for cell viability and both are required for invasive filamentous growth [6], [23]. Here we investigated the dynamics of *C. albicans* Rac1 using FRAP and FLIP approaches, together with the importance of its carboxyl-terminal region for its function and localization.

## Materials and Methods

### Growth conditions

Yeast extract-peptone dextrose (YEPD) or synthetic complete (SC) medium was used, and strains were grown at 30°C, unless indicated otherwise. Filamentous growth induction was carried out in liquid media containing 50% serum [24]. Filamentous growth induction in embedded media was carried out in YEP containing 2% sucrose and 2% agar [25].

### Strains and plasmids

Strains used in this study are listed in Table 1. To generate complemented or over-expression strains, the pExpArg-derived plasmids [6] were digested with StuI, and targeted to the *RP10* locus in BWP17 [26], *cdc42Δ/pMETCDC42* [24] or *rac1Δ/rac1Δ* [6]. Two independent clones of each strain were generated.

**Table 1:** Yeast strains used in this study.

Strain	Relevant genotype	Reference
BWP17	*ura3*Δ*::λimm434/ura3*Δ*::λimm434 his1*Δ*::hisG/his1*Δ*::hisG arg4*Δ*::hisG/arg4*Δ*::hisG*	[26]
PY189	*rac1*Δ::*URA3/rac1*Δ::*HIS1 arg4*Δ::*hisG/arg4*Δ::*hisG*	[6]
PY191	Same as PY189 with *RP10*::*ARG4*	[6]
PY196	*cdc42Δ::HIS1/cdc42::URA3-PMET3CDC42* with *RP10*::*ARG4*-*PADH1GFPCDC42*	[6]
PY201	Same as BWP17 with *RP10*::*ARG4*-*PADH1GFPRAC1*	[39]
PY205	Same as PY189 with *RP10*::*ARG4*-*PADH1GFPRAC1*	[6]
PY209	Same as PY189 with *RP10*::*ARG4*-*PADH1GFPrac1[G12V]*	[6]
PY212	Same as PY189 with *RP10*::*ARG4*-*PADH1GFPrac1[T17N]*	[6]
PY225	*cdc42Δ::HIS1/cdc42::URA3-PMET3CDC42* with *RP10*::*ARG4*-*PADH1GFPRAC1*	This study
PY250	*cdc42Δ::HIS1/cdc42::URA3-PMET3CDC42* with *RP10*::*ARG4*-*PADH1GFPCDC42CT_RAC1_*	This study
PY255	Same as PY189 with *RP10::ARG4-PADH1GFPCDC42*	This study
PY257	Same as PY189 with *RP10::ARG4-PADH1GFPCDC42CT_RAC1_*	This study
PY275	Same as PY189 with *RP10::ARG4-PRAC1RAC1*	[6]
PY353	Same as PY189 with *RP10*::*ARG4-PADH1GFPRAC1CT_CDC42_*	This study
PY357	Same as PY189 with *RP10::ARG4-PADH1GFPCT_RAC1_*	This study
PY406	Same as PY189 with *RP10::ARG4-PRAC1rac1[C233S]*	This study
PY415	Same as PY189 with *RP10::ARG4-PADH1GFPrac11[C233S]*	This study
PY438	Same as PY189 with *RP10::ARG4-PADH1GFPCT_RAC1[C233S]_*	This study
PY511	Same as PY189 with *RP10::ARG4-PADH1GFPrac1-5Q*	This study
PY534	Same as PY189 with *RP10::ARG4-PRAC1rac1-5Q*	This study
PY913	Same as PY189 with *RP10::ARG4-PADH1GFPHsRAC1*	This study
PY1222	Same as PY189 with *RP10::ARG4-PADH1GFPHsRACT_CaRAC1_*	This study
PY1265	*dck1Δ::HIS1/dck1Δ::URA3* with *RP10*::*ARG4*-*PADH1GFPRAC1*	[39]
PY1598	*rdi1Δ::HIS1/rdi1Δ::URA3* with *RP10*::*ARG4*-*PADH1GFPRAC1*	This study

To correct for codon usage in *C. albicans* [27], the 7 CTG codons of *HsRAC1*, coding for Leu20, Leu53, Leu129, Leu134, Leu155, Leu189 and Leu190 were mutated. Specifically, *HsRAC1* was amplified by PCR from *pKH3HARAC1* [28], using gene specific primers, with a unique RsrII site 5′ of the ATG and a unique MluI site 3′ of the stop codon and the Leu189 and Leu190 codons altered, respectively. This PCR product was cloned into pCR2.1 TA (Invitrogen, Cergy Pontoise, France), yielding pCR-*HsRAC1*. Subsequently, the 5 additional Leu codons were modified by site directed mutagenesis, using primer pairs containing unique restriction sites to facilitate mutant's identification, *i.e.* AccI (Leu20), AvaI (Leu53), HindIII (Leu129, Leu134), and XhoI (Leu155). Furthermore, the base at position 489 was modified to remove a StuI restriction site, which was used to integrate the plasmid at the *RP10* locus. The resulting plasmid was then digested by RsrII and MluI to release the mutated *HsRAC1* fragment, which was cloned into the respective sites in pExp-*PADH1GFP* [23], yielding pExp-*PADH1GFPHsRAC1*. HsRac1CT_CaRac1_ chimera resulted from the replacement of the region encoding the last carboxyl-terminal 12 residues of *HsRAC1* by that of the last carboxyl-terminal 14 residues of *CaRAC1*.

Mutation of the 2 potential nuclear localization sequences at the C-terminus of Rac1 (*rac1-5Q*) was achieved by site-directed mutagenesis on pExp-*PADH1GFPRAC1*, resulting in the mutation of K_223_KRKIKR_229_ to Q_223_QQKIQQ_229_. Similarly, pExp-*PADH1GFPrac1[C233S]* was generated by site-directed mutagenesis, using gene specific primers, containing a unique ScaI site to facilitate mutant's identification. All pExp-*PADHGFPX* constructs had a MluI site 3′ of *RAC1* stop codon and contained an endogenous EcoRV site 60 bp 3′ of *RAC1* ATG. EcoRV-MluI fragments from the different constructs were sub-cloned into the respective sites in pExp-*PRAC1RAC1*, yielding pExp-*PRAC1rac1-5Q* and pExp-*PRAC1rac1[C233S]*.

To generate Rac1CT_Cdc42_ and Cdc42CT_Rac1_ chimeras, we used PCR amplification of either *RAC1* or *CDC42*, respectively, with a primer containing a unique RsrII site 5′ of the respective ATG and a primer which anneals to this same gene and contains sequence encoding for the carboxyl-terminal tail of the gene of interest followed by a unique MluI site 3′ of the stop codon. This amplified DNA fragment was subsequently cloned into the respective sites in pExp-*PADH1GFP*. Rac1CT_Cdc42_ chimera resulted from the replacement of the region encoding the last carboxyl-terminal 14 residues of *RAC1* by that of the last carboxyl-terminal 12 of *CDC42*. The reciprocal Cdc42CT_Rac1_ chimera resulted from the replacement of the region encoding the last carboxyl-terminal 12 residues of *CDC42* by that of the last carboxyl-terminal 14 of *RAC1*. GFP-CT_Rac1_ and GFP-CT_Rac1[C233S]_ chimeras resulted from the fusion of the sequence encoding the carboxyl-terminal 14 residues of either *RAC1* or *rac1*[C233S] to *GFP*. *GFP* was amplified by PCR, using a primer containing a unique RsrII site 5′ of the ATG and a primer which annealed to the 3′ end of GFP (lacking the stop codon) followed by the carboxyl-terminal portion of *RAC1* or *rac1[C233S]*, a stop codon and an MluI site.

The sequence of all cloned PCR products and site directed mutagenesis products were confirmed (Eurofins MWG Operon, Ebersberg Germany and Seqlab - Sequence Laboratories, Göttingen, Germany).

### Microscopy

Colonies and cells were imaged as described previously [6]. Fluorescence recovery after photobleaching (FRAP) analysis was performed essentially as described previously [6] using a Zeiss LSM510 Meta confocal microscope with a Plan-Apo 63× (numerical aperture, 1.4) objective. For the majority of plasma membrane FRAP experiments, images were captured every 0.2–0.4 sec at 2–5% maximum laser intensity and 10×0.25–0.5 ms photobleaching scans at 100% laser intensity were performed on a circular area of 1 µm^2^ at the bud tip. For plasma membrane FRAP experiments with GFP-Rac1[T17N] expressing cells, conditions were identical except images were captured every 2.5 sec and 10×1 ms photobleaching scans were used. For nuclear FRAP experiments, images were captured every 2–5 sec at 3–6% maximum laser intensity and 8–12×1–1.2 ms photobleaching scans at 100% laser intensity were performed on a circular area of 1 µm^2^ within the center of the nucleus. Data analysis was carried out as described [6]. For plasma membrane FLIP experiments, images were captured every 4 sec at 4% laser intensity and 10×0.32 ms photobleaching scans at 100% laser intensity were performed on a circular area of 1 µm^2^ covering the plasma membrane. Photobleaching was repeated after every 4 images.

### General techniques

For analyses of colony morphology, plates were incubated for 5 days and images of colonies were taken with a Roper Scientific Micromax CCD camera on a Leica MZ6 dissection scope at a magnification of 10×. For cell morphology studies, cells were imaged with a CCD camera on a Leica DMR epifluorescence microscope with a numerical aperture 1.35 63× objective or with a numerical aperture 1.4 100× by differential interference contrast (DIC) optics, as described [24]. For Western blots, pellets of logarithmically growing cells (OD_600_∼1) were resuspended in SDS-polyacrylamide gel electrophoresis (PAGE) sample buffer, and analyzed by 10% SDS-PAGE, followed by immunoblot analyses. Proteins on nitrocellulose membranes were initially visualized by Ponceau S staining. Blots were then probed with a rabbit polyclonal serum against GFP (1∶5000; [29] and visualized by enhanced chemiluminescence (luminol-coumaric acid) on a Fuji-Las3000 (Clichy, France).

## Results

### *C. albicans* Rac1 accumulates in the nucleus

**Figure 1:**
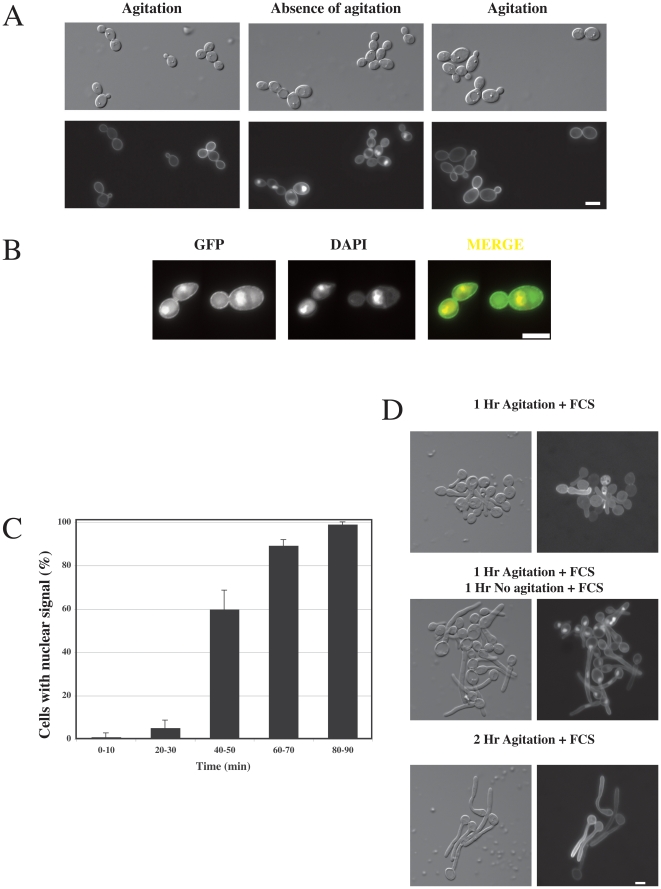
*C. albicans* Rac1 accumulates in the nucleus. (A) Intracellular accumulation of GFP-Rac1 in the absence of cell agitation. DIC and fluorescence images of *rac1*Δ*/rac1*Δ *PADH1GFPRAC1* (PY205) cells from shaking cultures, after 1 h without agitation, followed by 1 h of shaking. Bar, 5 µm. (B) Rac1 accumulates in the nucleus in the absence of cells agitation. Intracellular GFP-Rac1 co-localizes with 4′,6-diamidino-2-phenylindole (DAPI). (C) Time-course of Rac1 nuclear accumulation. Cells with nuclear fluorescent signal were counted after the indicated times in the absence of agitation. The average of four experiments is shown with approximately 50 cells counted at each time point in each experiment (bars indicate standard deviation). (D) Rac1 accumulates in the nucleus of serum-induced hyphal cells. Hyphal growth in PY205 cells was induced in rich (YEPD) liquid media containing 50% FCS, for the indicated times, in the presence or the absence of agitation at 37°C.

In mammalian cells, Rac1 accumulates in the nucleus [12], [13] where it is proposed to have different functions, such as activating proteins of the STAT family [11] or promoting cell division [10]. Intriguingly, in *C. albicans* we observed that when cells sediment, Rac1 also accumulates in an organelle, which is likely to be the nucleus. Specifically, we observed that while GFP-Rac1 localized to the plasma membrane in exponentially growing cells under agitation (Figure 1A, left panel), this fusion protein accumulated in an intracellular compartment in the absence of agitation (Figure 1A, middle panel). If these cells were then subsequently agitated, accumulation in this intracellular compartment was no longer observed (Figure 1A, right panel). To confirm that Rac1 is indeed in the nucleus, we examined GFP fluorescence in cells grown in low levels of 4′,6-diamidino-2-phenylindole (DAPI), which labels DNA. The GFP and DAPI signals co-localized as illustrated in Figure 1B. When the nuclear accumulation of Rac1 was followed over time, we observed ∼100% cells with nuclear fluorescence after 80–90 min in the absence of agitation (Figure 1C). Rac1 nuclear accumulation was independent of the cell cycle stage and of the culture media conditions, including the presence of serum, which induces hyphal growth (Figure 1D).

**Figure 2:**
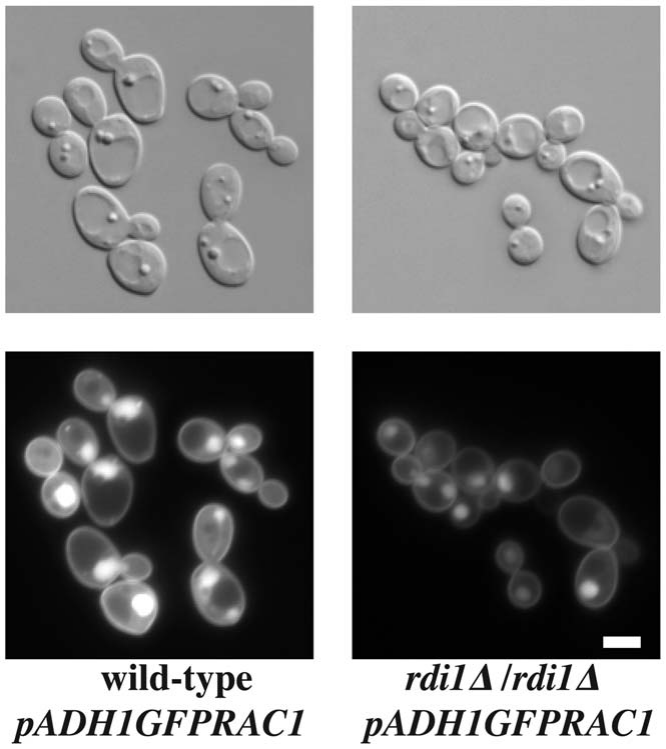
Rdi1 is not necessary for *C. albicans* Rac1 nuclear accumulation. DIC and fluorescence images of wild-type cells with *PADH1GFPrac1* (PY201) and *rdi1Δ/rdi1Δ PADH1GFPrac1* (PY1598) cells were taken after 75 min without agitation. Bar, 5 µm.

In mammals, Rac1 localization is regulated by RhoGDI [9]: RhoGDI sequesters the GDP-bound GTPase in the cytoplasm, keeping it as a source of activatable GTPase, [30] or shuttling it to other membranes within the cell [31]. In *S. cerevisiae*, the sole RhoGDI, Rdi1, interacts with Cdc42, Rho1 and Rho4 [32], [33]. Rdi1 has also been shown to play a role in Cdc42 fast plasma membrane recycling [34]. In *C. albicans*, Rdi1 also interacts with Cdc42, but deletion of *RDI1* had little to no consequences, only altering filamentous growth when coupled with deletions of the Cdc42 GTPase-activating proteins Rga2 and Bem3 [35]. In our conditions, Rac1 nuclear accumulation was still observed in a *rdi1* deletion mutant (Figure 2), indicating that Rdi1 is not required for such a process.

**Figure 3:**
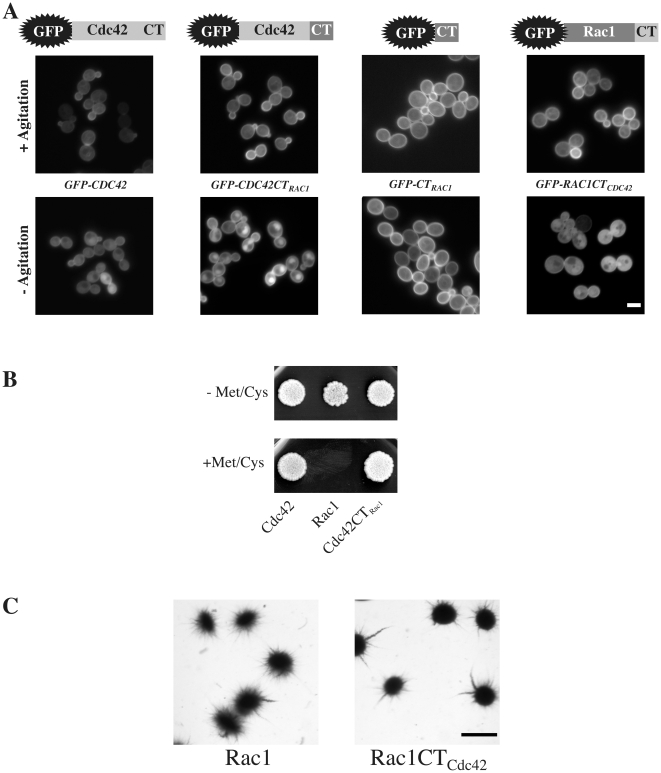
Rac1 carboxyl-terminal region is necessary but not sufficient for nuclear targeting. (A) Fluorescence images of indicated strains (PY255, PY257, PY357 and PY353) expressing GFP-Cdc42, GFP-Cdc42CT_Rac1_, GFP-CT_Rac1_ and GFP-Rac1CT_Cdc42_. Images of cells from shaking cultures(+ Agitation) or after 1 h in the absence of agitation (− Agitation) are shown. Schematic representations of the chimeras are illustrated above. Bar, 5 µm. (B) Over-expression of Cdc42CT_Rac1_ complements for viability of *cdc42* mutant cells. Exponentially growing *cdc42Δ/pMETCDC42* over-expressing *GFPCDC42* (PY198), *GFPRAC1* (PY225), and *GFPCDC42CT_RAC1_* (PY250) cells were spotted on selective media containing agar, in the presence or the absence of 2.5 mM methionine and cysteine [24] and grown for 3 days at 30°C. (C) Over-expression of Rac1CT_Cdc42_ complements the embedded matrix-dependent filamentous growth defect of *rac1* mutant cells. Cells from *rac1*Δ*/rac1*Δ *PADH1GFPRAC1* (PY205) and *rac1*Δ*/rac1*Δ *PADH1GFPRAC1CT_CDC42_* (PY353) were embedded in YEPS as described [25] and images of colonies were taken after 5 days at 25°C. Similar results were observed in two independent experiments. Bar, 1 mm.

While Rac1 accumulates in the nucleus, such a nuclear localization was not observed for another Rho G-protein, Cdc42, which has 60% overall sequence identity with Rac1 (Figure 3A); the clustered membrane localization of Cdc42 at the bud tip was, however, somewhat less pronounced in the absence of agitation. A chimera in which the carboxyl-terminal 12 residues of Cdc42 replaced the 14 carboxyl-terminal residues of Rac1 (Rac1CT_Cdc42_) did not substantially accumulate in the nucleus in these conditions. In contrast, the converse chimera (Cdc42CT_Rac1_) accumulated in the nucleus, in the absence of agitation. The same 14 residues carboxyl-terminal region of Rac1 fused to GFP (GFP-CT_Rac1_) did not result in GFP nuclear accumulation in the absence of cell agitation (Figure 3A). In *C. albicans*, Cdc42 is in particular required for cell viability [36], [37], while Rac1 is required for invasive filamentous growth in solid media including when cells are embedded in a matrix [6], [23]. Cdc42CT_Rac1_ was functional, when expressed as the sole Cdc42 copy, for viability (Figure 3B). Similarly, Rac1CT_Cdc42_ was functional, when expressed as a sole Rac1 copy, for embedded filamentous growth, suggesting that Rac1 nuclear accumulation is not required for embedded filamentous growth (Figure 3C). All together, our results indicate that *C. albicans* Rac1 can accumulate in the nucleus in the absence of cell agitation. The Rac1 carboxyl-terminal region is necessary for this process, yet not sufficient to target a heterologous protein such as GFP to the nucleus.

### Rac1 carboxyl-terminal region is required for localization and function

**Figure 4:**
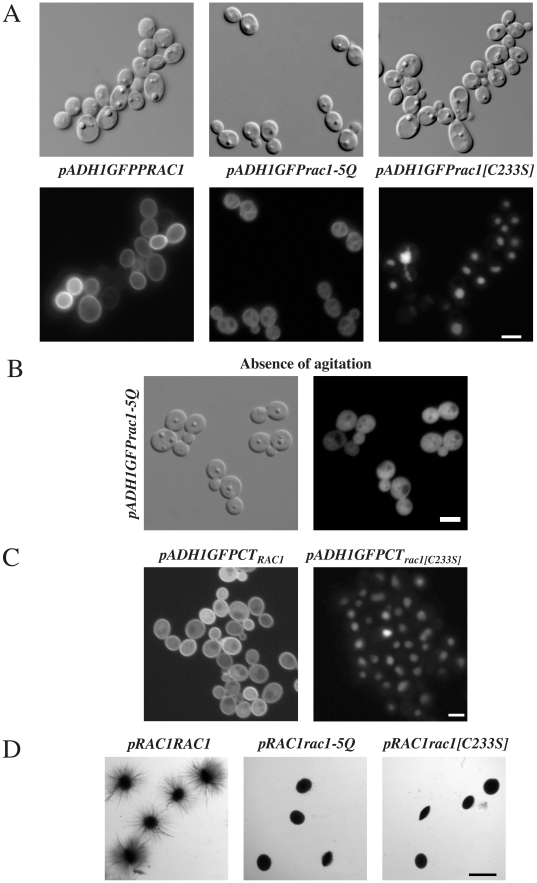
Rac1 polybasic carboxyl-terminal region is required for membrane localization and function. (A) Rac1 PBR is required for plasma membrane localization. DIC and fluorescence images of indicated strains *rac1*Δ*/rac1*Δ *PADH1GFPRAC1* (PY205), *rac1*Δ*/rac1*Δ *PADH1GFPrac1-5Q* (PY511), and *rac1*Δ*/rac1*Δ *PADH1GFPrac1[C233S]* (PY415), respectively, are shown. Bar, 5 µm. (B) Rac1 PBR is required for nuclear accumulation. DIC and fluorescence images of *rac1*Δ*/rac1*Δ *PADH1GFPrac1-5Q* (PY511) incubated 75 min without agitation are shown. Bar, 5 µm. (C) In the absence of prenylation, the Rac1 carboxyl-terminal region targets GFP to the nucleus. Fluorescence images of indicated strains (PY357 and PY438, respectively) are shown. Bar, 5 µm. (D) Rac1 PBR is required for function. Cells from *rac1*Δ*/rac1*Δ *PRAC1RAC1* (PY275), *rac1*Δ*/rac1*Δ *PRAC1rac1-5Q* (PY534), and *rac1*Δ*/rac1*Δ *PRAC1rac1[C233S]* (PY406) were embedded in YEPS and images of colonies were taken, as described in Figure 3C. Similar results were observed in 3 independent experiments. Bar, 1 mm.

In human cells, the Rac1 carboxyl-terminal region has been shown to be important both for localization and function. In particular, a nuclear localization sequence (NLS) in the HsRac1 carboxyl-terminal polybasic region (PBR) was identified and shown to be required for nuclear accumulation [12], [13]. Based on the consensus NLS motif (K-K/R-X-K/R), we identified two potential NLS's in *C. albicans* Rac1 carboxyl-terminal PBR: K_225_KRK_228_ and K_230_RAK_233_. To investigate the role of these sequences in Rac1 sub-cellular localization, we initially generated strains expressing GFP-Rac1-5Q (Q_225_QQKIQQ_231_, replacing K_225_KRKIKR_231_). We also generated a strain expressing GFP-Rac1[C233S], which cannot be geranylgeranylated. Figure 4A shows that GFP-Rac1-5Q was not observed at the plasma membrane and instead was present in the cytoplasm as well as associated with internal membranes. This Rac1-5Q mutant did not accumulate in the nucleus in the absence of cell agitation (Figure 4B). In contrast, GFP-Rac1[C233S] accumulated predominantly in the nucleus (Figure 4A) in the same condition. In addition, while GFP fused to the 14 carboxyl-terminal residues of Rac1 (GFP-CT_Rac1_) did not accumulate in the nucleus in the absence of cell agitation (Figure 3A), mutation of Cys at position 233 to Ser (GFP-CT_Rac1[C233S]_) resulted in nuclear accumulation of this fusion (Figure 4C). These results indicate that the NLS motifs present in the PBR are necessary and sufficient for targeting Rac1 to the nucleus in *C. albicans*, and suggest that the function of these NLS sequences is blocked or overridden by geranylgeranyl modification, as has been proposed for mammalian cells [10]. As expected if Rac1 functions primarily at the plasma membrane, *rac1-5Q* and *rac1[C233S]* were unable to restore embedded filamentous growth in a *rac1* deletion mutant (Figure 4D). Together, these results indicate that the carboxyl-terminal polybasic region of *C. albicans* Rac1, similar to that of HsRac1, is necessary for its localization and function.

### Rac1 cycles in and out of the nucleus

**Figure 5:**
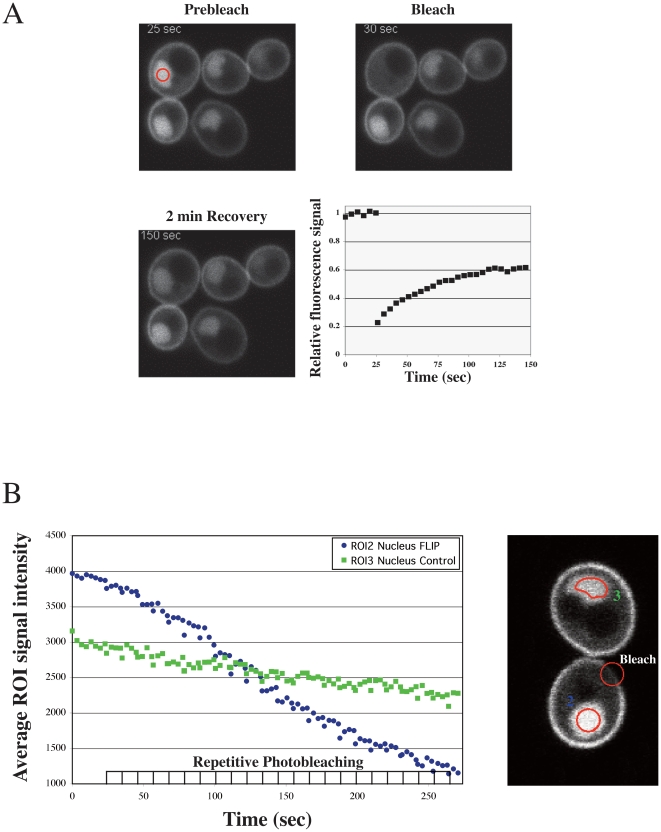
Rac1 cycles in and out of the nucleus. (A) FRAP analysis of nuclear Rac1. Confocal microscopy images of budding *rac1*Δ*/rac1*Δ *PADH1GFPRAC1* (PY205) cells, after 1 h in the absence of agitation, were taken prior to and subsequent to photobleaching of the nucleus. A typical nuclear fluorescence recovery after photobleaching experiment is shown. (B) FLIP analysis of nuclear Rac1. Confocal microscopy images of budding PY205 cells, after 1 h in the absence of agitation, were taken subsequent to photobleaching a region on the plasma membrane (Bleach). For fluorescence loss in photobleaching experiments, images of the nucleus of the photobleached cell (ROI2 nucleus FLIP) together with images of the nucleus of an adjacent cell (ROI3 nucleus control) were captured every 4 sec. Plasma membrane photobleaching was repeated after every 4 images.

To determine if *C. albicans* Rac1 can undergo nucleo-cytoplasmic shuttling, we investigated Rac1 dynamics using both fluorescence recovery after photobleaching (FRAP) and fluorescence loss in photobleaching (FLIP) [38] approaches. In the former approach, the nuclear fluorescence was photobleached and the time-course of fluorescence recovery (FRAP t_½_) was determined. Figure 5A illustrates a typical experiment in which a portion of the nucleus (indicated by a circle) was bleached. This resulted in the loss of the nuclear fluorescence signal, consistent with the notion that the Rac1 nuclear pool is freely diffusible. The average FRAP t_½_ for nuclear GFP-Rac1 was 34.21±12.02 sec (*n* = 25), indicating that Rac1 is imported into the nucleus on the timescale of a minute. In the FLIP approach, a portion of the plasma membrane of cells in which GFP-Rac1 has accumulated in the nucleus was repetitively bleached, and the intensity of the nuclear fluorescence signal was followed over time (Figure 5B). The intensity of the nuclear fluorescence signal decreased faster in the cell in which the plasma membrane was repetitively bleached compared to an adjacent control cell (Figure 5B), suggesting that the nuclear and plasma membrane pools of Rac1 are connected. The results from these different photo-bleaching experiments indicate that Rac1 cycles in and out of the nucleus.

### Rac1 dynamics depend on its activation state

**Figure 6:**
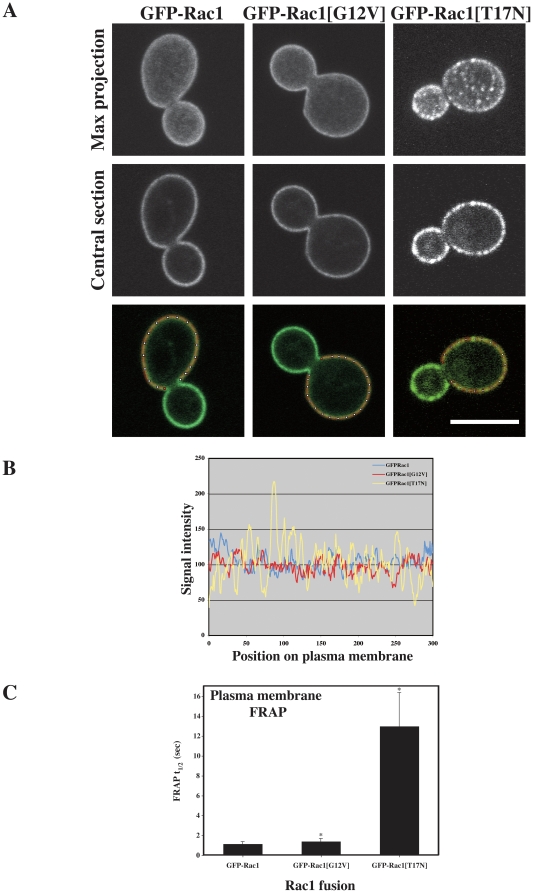
Rac1 dynamics at the plasma membrane depend on its activation state. (A) Localization of Rac1, activated (Rac1[G12V]) or inactivated (Rac[T17N]) forms. Confocal microscopy images of budding *rac1*Δ*/rac1*Δ *PADH1GFPRAC1* (PY205), *rac1*Δ*/rac1*Δ *PADH1GFPrac1[G12V]* (PY209), and *rac1*Δ*/rac1*Δ *PADH1GFPrac1[T17N]* (PY212) cells were taken; both the maximal projection and central section are shown. (B) Inactivated and activated forms of Rac1 have different plasma membrane distributions. Graph of signal intensity along plasma membrane perimeter of the indicated cells, as shown in the bottom panel of Figure 6A. (C) FRAP analysis of cells expressing Rac1 (*n* = 17), its activated (Rac1[G12V]) (*n* = 20) or inactivated (Rac[T17N]) (*n* = 17) forms. FRAP t_½_ values (means ± standard deviation) are determined from single-phase exponential curve fit of fluorescence recovery after photobleaching. The two-tailed P-values of the indicated mean FRAP t_½_'s (*) are less than 0.006 compared with the FRAP t_½_ of GFP-Rac1.

**Figure 7:**
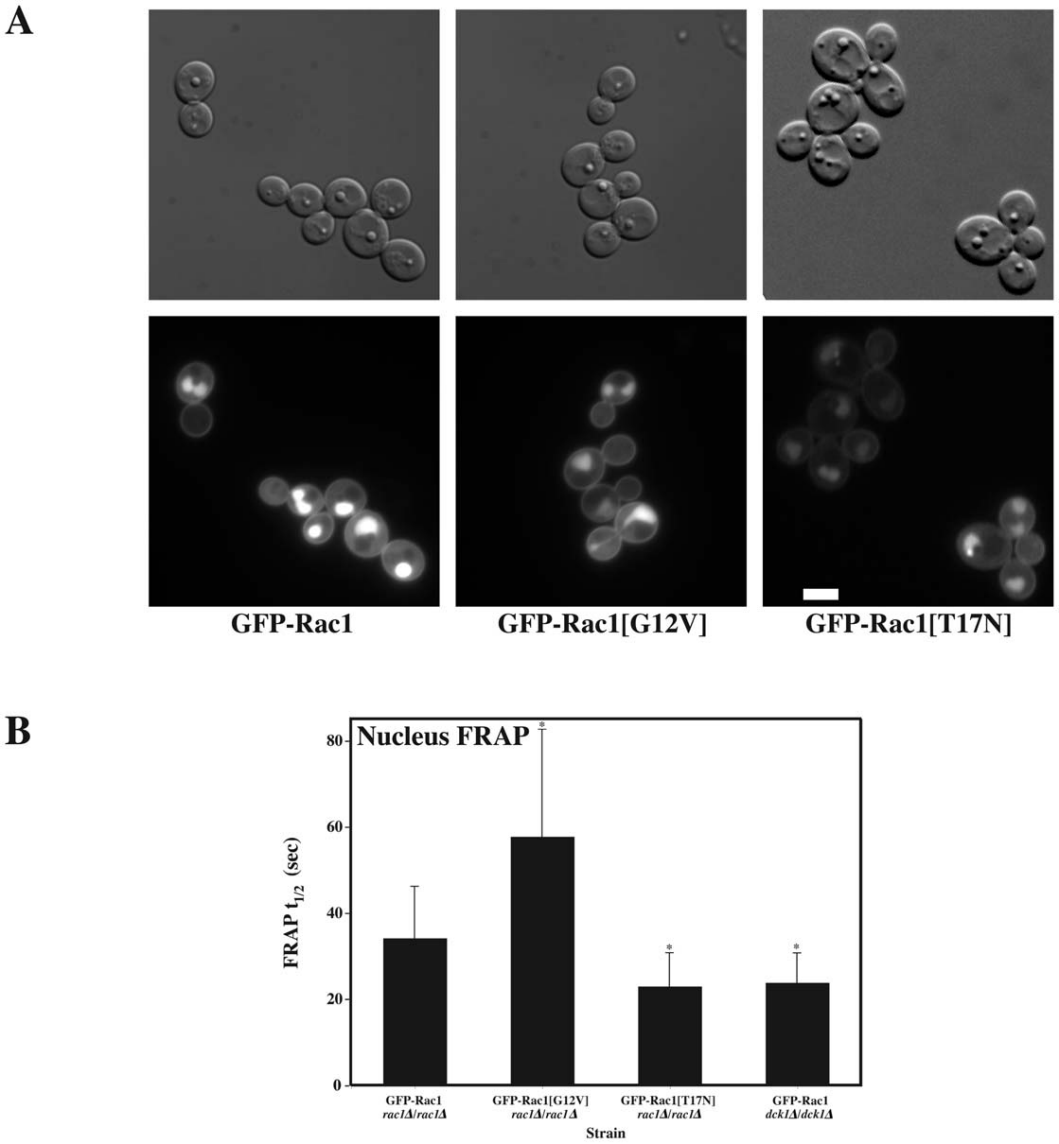
Rac1 dynamics in the nucleus depend on its activation state. (A) Nuclear localization of Rac1 depends on its activation state. DIC and fluorescence images of budding *rac1*Δ*/rac1*Δ *PADH1GFPRAC1* (PY205), *rac1*Δ*/rac1*Δ *PADH1GFPrac1[G12V]* (PY209), and *rac1*Δ*/rac1*Δ *PADH1GFPrac1[T17N]* (PY212) cells after 1 h in the absence of agitation were taken. Bar, 5 µm. (B) Rac1 dynamics at the nucleus depend on its activation state. FRAP analysis of nucleus of *rac1*Δ*/rac1*Δ *PADH1GFPRAC1* (PY205), (*n* = 25), *rac1*Δ*/rac1*Δ *PADH1GFPrac11[G12V]* (PY209), (*n* = 23), *rac1*Δ*/rac1*Δ *PADH1GFPrac11[T17N]* (PY212) (*n* = 21), and *dck1*Δ*/dck1*Δ *PADH1GFPRAC1* (PY1265) (*n* = 30) cells, after 75–90 min in the absence of agitation. FRAP t_½_ values (means ± standard deviation) are determined from single-phase exponential curve fits of FRAP intensities, as described Figure 5A. The two-tailed P-values of the indicated mean FRAP t_½_'s (*) are less than 0.0005 compared with the FRAP t_½_ of GFP-Rac1.

**Figure 8:**
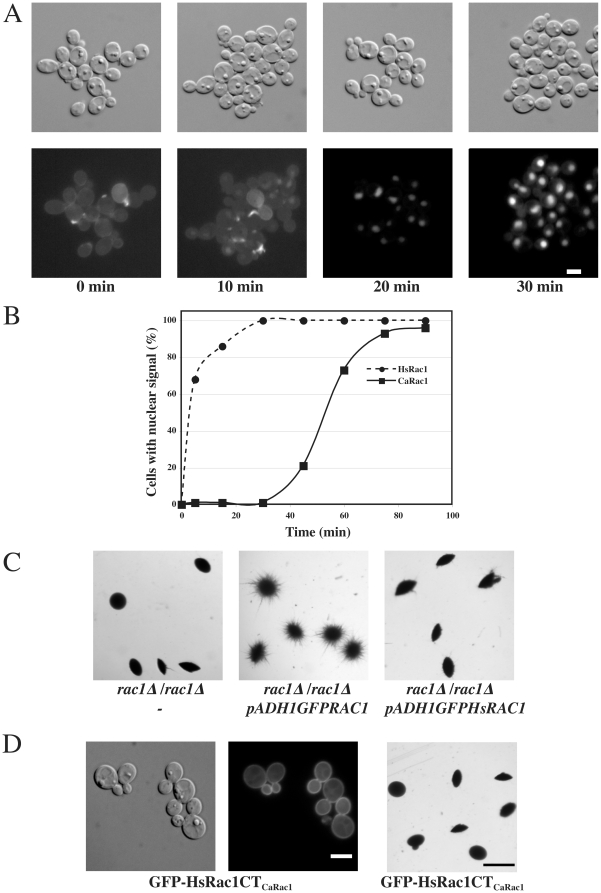
Nuclear accumulation is an inherent property of Rac1. (A) Human Rac1 (HsRac1) accumulates in the nucleus of *C. albicans* cells. *HsRAC1* was corrected for codon usage in *C. albicans* (see Materials and methods). DIC and fluorescence images of *rac1*Δ*/rac1*Δ *PADH1GFPHsRAC1* (PY913) cells were taken after the indicated times without agitation. Bar, 5 µm. (B) Time-course of HsRac1 nuclear accumulation. Cells expressing GFP-HsRac1 (PY913) and GFP-CaRac1 (PY205) with nuclear fluorescent signal were counted after the indicated times in the absence of agitation (*n* = 100–200 cells for each time point). (C) HsRac1 cannot complement for CaRac1 function. Cells from *rac1*Δ*/rac1*Δ (PY191), *rac1*Δ*/rac1*Δ *PADH1GFPRAC1* (PY205), and *rac1*Δ*/rac1*Δ *PADH1GFPHsRAC1* (PY913), were embedded in YEPS as described [25] and images of colonies were taken after 5 d at 25°C. Similar results were observed in 3 independent experiments. Bar, 1 mm. (D) A chimera in which the 14 carboxyl terminal residues of CaRac1 replaced the 12 corresponding residues of HsRac1 (HsRac1CT_CaRac1_) localized to the plasma membrane, yet was not functional in embedded filamentous growth. Left two panels: DIC and fluorescence images of *rac1*Δ*/rac1*Δ *PADH1GFPHsRAC1CT_CaRAC1_* (PY1222) are shown. Bar, 5 µm. Right panel: PY1222 cells were embedded in YEPS and images of colonies were taken as in Figure 8C. Bar, 1 mm.

To address whether Rac1 dynamics depend on its activation state, we compared the localization and FRAP dynamics of Rac1 and its derivatives, which mimic either the activated form (Rac1[G12V]) or the inactivated form (Rac1[T17N]). Figure 6A illustrates the localization of GFP-Rac1, GFP-Rac1[G12V] or GFP-Rac1[T17N] in exponentially growing cells, showing both the maximal projections and the central optical sections. We observed that Rac1 and Rac1[G12V] were distributed similarly at the plasma membrane. In contrast, Rac1[T17N] was localized to clusters at the plasma membrane (Figures 6A and 6B). The FRAP t_½_ of Rac1[G12V] was slightly slower compared to Rac1 (1.41±0.99 sec and 1.15±0.23 sec, respectively), whereas that of Rac1[T17N] was substantially slower (13.00±3.38 sec) (Figure 6C). During the time course of the FRAP experiments (∼2 min) we did not observe movement of the Rac1[T17N] clusters at the plasma membrane. These results indicate that Rac1[T17N] is substantially less mobile at the plasma membrane than Rac1 or Rac1[G12V], and suggest that the rapid dynamics of this Rho G-protein at the plasma membrane requires GTPase cycling. Similarly, we previously showed that the fluorescence recovery of GFP-Rac1 was slower in cells lacking the Rac1 specific activator, Dck1, or the scaffold protein Lmo1 [39]. Furthermore, we examined whether these different mutant forms of Rac1 could accumulate in the nucleus. Figure 7A shows that both Rac1[G12V] and Rac1[T17N] can accumulate in the nucleus, indicating that GTPase cycling is not required for this nuclear import. Compared to the FRAP measurements of nuclear Rac1 (t_½_ = 34.21±12.02 sec), Rac1[G12V] had a significantly slower recovery of nuclear fluorescence (t_½_ = 57.82±24.86 sec) and Rac1[T17N] a significantly faster recovery of nuclear fluorescence (t_½_ = 23±6.8 sec) (Figure 7B). Furthermore, although there was no significant difference between t_½_ recovery of nuclear fluorescence of Rac1 in wild-type cells (t_½_ = 35.54±11.70 sec, *n* = 18) and *rac1*Δ*/rac1*Δ cells (t_½_ = 34.21±12.02 sec, *n* = 25), the t_½_ recovery of nuclear fluorescence of Rac1 in *dck1*Δ*/dck1*Δ cells was significantly faster (t_½_ = 23.88±7.73 sec, *n* = 30; Figure 8B), consistent with the results from the *rac1[T17N]* strain. These data suggest that while active and inactive forms of Rac1 can accumulate in the nucleus, the latter is imported into the nucleus roughly 2.5 times faster than the former. Together these results show that Rac1 dynamics both at the plasma membrane as well as shuttling in and out of the nucleus depend on the activation state of this G-protein.

### Nuclear accumulation is an inherent property of Rac1

As both human Rac1 and *C. albicans* Rac1 can accumulate in the nucleus, we investigated the heterologous expression of human Rac1 in *C. albicans*. Specifically, we expressed in *C. albicans* human Rac1 (HsRac1), corrected for codon usage and examined its localization in cells collected at different times without agitation (Figure 8A). In cells taken immediately from a shaking culture we observed HsRac1 in the cytoplasm and on the plasma membrane. In the absence of agitation HsRac1 rapidly accumulated into the nucleus. Indeed HsRac1 accumulated in nuclei faster than CaRac1, with ∼70% of the HsRac1 expressing cells exhibiting nuclear fluorescence after 5 min in the absence of agitation compared to CaRac1 expressing cells, in which similar nuclear accumulation was observed only after 60 min (Figure 8B). Furthermore, as illustrated in Figure 8C, HsGFPRac1 did not restore invasive filamentous growth in a *rac1* deletion strain, compared to a control strain expressing the *C. albicans RAC1* GFP fusion. We verified that the fusion proteins migrated at the expected size on SDS-PAGE, by immunoblot using anti-GFP sera. One possibility why HsRac1 does not complement for the function of *C. albicans* Rac1 is that this protein was less efficiently targeted to the plasma membrane. Hence we generated a chimera, HsRac1CT_CaRac1,_ in which the last carboxy-terminal 12 residues of HsRac1 were replaced by the last 14 carboxl-terminal residues of CaRac1. As illustrated in Figure 8D, this chimera localized to the plasma membrane, yet was still unable to complement the *C. albicans* embedded filamentous growth defect of a *rac1* deletion strain, making it unlikely that inefficient membrane targeting can explain HsRac1 inability to function in embedded growth. Nonetheless, our results indicate that nuclear accumulation is an inherent property of Rac1 and suggest the existence of a fundamental mechanism for Rac1 nuclear import and retention.

## Discussion

In human cells, Rac1 can localize to the nucleus [12], [13]. In this report, we show that *C. albicans* Rac1 can also accumulate in the nucleus. Our results indicate that the NLS-consensus motifs in the *C. albicans* Rac1 carboxyl-terminal region are required for targeting to the nucleus. Geranylgeranylation of the adjacent cysteine residue at position 233 blocks nuclear accumulation as Rac1[C233S] was only observed in the nucleus under all conditions examined. Similar results have been observed with human Rac1 [10], [12], [13]. These results indicate that Rac1 nuclear accumulation is an inherent property of this G-protein.

*C. albicans* Rac1 nuclear accumulation is dependent on the carboxyl-terminal portion of Rac1, as swapping the carboxyl-terminus of Rac1 for that of Cdc42 impaired nuclear accumulation, yet this carboxyl-terminal portion of Rac1 is not sufficient to target GFP to the nucleus in the absence of cell agitation. These results indicate that the guanine nucleotide binding part of the G-protein is also important for nuclear accumulation. As our results from FRAP and FLIP approaches indicate that Rac1 can cycle in and out of the nucleus, we envision that this G-protein must first be extracted from the plasma membrane prior to transport to the nucleus. While RhoGDIs have been reported to fulfill such a function [9] our results indicate that deletion of the sole RhoGDI in *C. albicans*, Rdi1, did not block Rac1 nuclear accumulation, suggesting that extraction of Rac1 from the plasma membrane might require another factor. Very recently, Sandrock *et al.* [40] reported that in HeLa cells, Rac1 nuclear import depends on karyopherin α2, which the authors propose interacts directly with Rac1. A homolog of this protein, importin α Srp1, is present in *S. cerevisiae* as well as in *C. albicans*, where its function has not yet been characterized. An attractive possibility would be that Rac1 is imported into the nucleus *via* interaction with Srp1. When expressed in *C. albicans* HsRac1 also accumulates in the nucleus, suggesting that the mechanism of Rac1 nuclear import and retention is conserved from yeast to human.

Rac1 nuclear localization in human cells is important for different functions, such as cell cycle regulation: Rac1 accumulates in the nucleus in late G2 and is excluded from this compartment in early G1 [10]. Our results indicate that in Rac1 accumulation in the nucleus in the absence of cell agitation can occur at all cell cycle stages in *C. albicans*, irrespective of whether cells are budding or undergoing filamentous growth. Furthermore, a Rac1-CT_Cdc42_ chimera that does not accumulate in the nucleus, is still functional for embedded filamentous growth. While we cannot rule out that this chimera undergoes rapid nuclear-cytoplasmic shuttling and hence does not accumulate appreciably in this organelle, these results suggest that the observed nuclear accumulation of Rac1 is not required for invasive filamentous growth. The kinetics of *C. albicans* Rac1 nuclear accumulation indicate that this is a slow process, as we begin to detect Rac1 fluorescent signal in the nucleus after ∼40 min in the absence of cell agitation. Given that the dynamics of Rac1 both at the membrane and in the nucleus are on the time scale of minutes, it is unlikely that cycling in and out of the nucleus is rate limiting for nuclear accumulation. Rac1 nuclear accumulation occurs in different media, including rich media, suggesting that nutrient limitation is not the trigger. An attractive possibility would be that Rac1 nuclear accumulation is triggered by an alteration in either oxygen or carbon dioxide levels upon cell sedimentation, however the function of Rac1 in the nucleus remains to be elucidated.

The nucleotide state of Rac1 in the nucleus could provide insight into its function in this organelle. Different studies have addressed the nucleotide state of human Rac1 in the nucleus. Using GFP fusions, it was shown that in CHO-m3 cells a constitutive active Rac1 mutant was slightly more efficiently targeted to the nucleus [12], [13], in contrast to MDCK and COS-1 cells in which a constitutive active GFP-Rac1 mutant had a similar distribution as GFP-Rac1 [10]. It was also shown that a constitutive active Rac1 mutant strongly accumulates in the nucleus of HeLa cells [40]. In contrast, studies using fluorescence resonance energy transfer (FRET)-based biosensors in Swiss 3T3 fibroblasts indicate that a large pool of GFP-Rac1 in the nucleus was inactive [17]. In *C. albicans*, we observed that both the constitutive active and inactive forms of Rac1 can accumulate in the nucleus in the absence of cell agitation. However, the FRAP t_½_ of nuclear Rac1[G12V] was significantly slower than that of Rac1. In contrast, nuclear Rac1[T17N] dynamics were significantly faster, similar to that of Rac1 in the absence of its activator Dck1. These results suggest that although both activated and inactivated mutants can accumulate in the nucleus, the inactive form of Rac1 accumulates faster in *C. albicans*. This could be the result of increased import, decreased export and/or the increase in a nuclear anchor. Our results are consistent with the notion that Rac1 nuclear accumulation serves to sequester this protein from the plasma membrane where it normally functions, potentially targeting it for degradation.
